# Off the Shelf Fouling Management

**DOI:** 10.3390/md15060176

**Published:** 2017-06-14

**Authors:** Daniel Rittschof

**Affiliations:** Nicholas School of the Environment, Duke University, Durham, NC 27708, USA; Ritt@duke.edu

**Keywords:** pharmaceuticals, fouling management, evolutionary principles, conserved pathways, metamorphosis, business models

## Abstract

This chapter tells the story of a research thread that identified and modified a pharmaceutical that could be a component of environmentally benign fouling management coatings. First, I present the background context of biofouling and how fouling is managed. The major target of the research is disrupting transduction of a complex process in all macrofouling organisms: metamorphosis. Using a bioassay directed approach we first identified a pharmaceutical candidate. Then, based on structure function studies coupled with laboratory and field bioassays, we simplified the molecule, eliminating halogens and aromatic rings to a pharmacophore that could be readily broken down by bacteria. Next, we did further structure function studies coupled to lab and field bioassays of modifications that enabled delivery of the molecule in a variety of coatings. The outcome is a different way of thinking about managing fouling and concepts in which molecules are designed to perform a function and then degrade. This work is discussed in the context of existing fouling management approaches and business models which use long-lived broad-spectrum biocides which have consequences for human, environmental health, and food security.

## 1. Introduction

This chapter is based upon work conducted by a large research team that I helped assemble and advised as a visiting research professor in Singapore from 2000 to 2015. The principals were Serena L-M Teo, James Maki, Christina Chai, Felicity Jameson, Chai Lung Chen, and Serina Lee Siew Chen. Others in the group can be found as authors on the publications the group generated. The story I tell is based upon my ideas. These ideas were brought to fruition by the team supported in Singapore by the Agency for Science, Technology and Research (A*STAR), Marine Port Authority (MPA), Economic Development Council (EDC), and other agencies listed in the publications. None of this story would have been possible without the transdisciplinary talents of all involved and the dedication and leadership of Serena Teo and Christina Chai. I provide this narrative in the hopes of providing perspective on many topics that researchers wishing to contribute to sustainable fouling management technology should consider. One might conclude that the title, Off the Shelf Fouling Management, is inadequate as a descriptor of the processes which are driven by the societal necessity to find sustainable management options.

There are necessary pressures to decrease non-target impacts of all essential environmental management approaches. The increase in human population coupled with economic globalization and inadequate resource management are fueling unprecedented threats to food security and human and environmental health. Societal and environmental change are so rapid that they could easily overwhelm the technological advances that enable our grand societies. Threats are a combination of many environmental stressors. Each remediated stressor helps us move towards a management strategy trajectory that is sustainable. Fouling management writ large is an environmental stressor.

## 2. Fouling and Fouling Management

Fouling management on ship hulls is a global environmental stressor because the existing management strategies employ long-lived broad-spectrum biocides [1,2]. The existing business models can be described as mix-and-kill strategies [3]. To gain perspective on fouling management and potential solutions, it helps to understand basic biofouling [1] because it is the phenomenon to be managed, for a historical perspective see references [3,4,5,6]. The future of fouling management could be reflected in changed regulations and business models which move away from the mix-and-kill strategies [3,7]. Rapidly developing economies which have antiquated low margin fouling management industries, no sophisticated coating, distribution, and application infrastructure, and no vested economic interest in maintaining unsustainable practices could take the lead with approaches that use up-to-date knowledge for sustainable fouling management. This chapter provides the theory for such an approach.

Fouling occurs at molecular, microbial, and macro-organismal levels. Biomolecules are the stuff of life. Microbes and macrobes are the generators of biomolecules responsible for molecular biofouling. Many biomolecules are amphipathic with regions compatible with hydrophilic and other regions compatible with lipophilic molecules. Amphipathic molecules are surfactants, meaning they are strongly surface active. There is a layer of active molecules on the surface of all natural waters, and biomolecules such as proteins, glycoproteins, proteoglycans, and phospholipids comprise sea foam, marine snow, and routinely coat all surfaces in aqueous environments. Biomolecules can be secreted on surfaces of organisms [8,9], generated by the action of extracellular enzymes called exoenzymes, or released upon the death of cells and organisms.

Biofouling, attachment to surfaces by living organisms, benefits the organisms by taking advantage of the flow of fluids which deliver nutrients and remove wastes. The ability to perform feeding and excretion with minimal expenditure of energy confers an advantage over organisms that must move large amounts of viscous water to perform nutrient acquisition and waste removal.

Microbial fouling by bacteria, cyanobacteria, algae, and diatoms can be passive or active. Some microbes settle like dust. Often microbes are associated with sticky extracellular polysaccharides which interact in the boundary layer over a surface and glue microbes to surfaces. Microbes on surfaces can form consortia. Consortia have emergent properties and can be considered as a different approach than classical metazoans to building a more complex organism [3].

Macrofouling is caused by plants and animals. Most animals that foul surfaces are filter feeders and consume phytoplankton and zooplankton. Macrofoulers become large and obvious and many have calcareous exoskeletons and can protrude tens of centimeters off a surface. Macrofouling on a surface like a ship hull dramatically increases drag, reduces performance, and increases costs. Macrofouling can be closely dependent on microbial fouling [10,11]. However, for many common macrofoulers around the globe, reports show bacterial films can have no effect, sometimes be stimulatory, and sometimes be inhibitory [12,13,14,15,16,17]. For comprehensive conversations on the spectrum of fouling possibilities, see [4,18,19].

All levels of fouling respond to surface properties such as surface energy. Molecules and microbes foul all surface energies [20,21,22,23,24]. In aqueous environments, adhesives work by excluding water, as reviewed in [25]. For strongly hydrophobic surfaces, it is easy to exclude water. For strongly hydrophilic surfaces, it is difficult to exclude water because a stable strongly hydrophilic surface is an excellent antifouling surface. One kind of hydrophilic material, zwiterionic material, is under development and is the topic of a 2016 special issue of Acta Biomaterialia, edited by Jiang et al. [26], also see [27]. Foul release surfaces are surfaces with intermediate surface energies like poly dimethyl siloxane which are weakly hydrophobic and do not provide opportunities for strong bonds to form [28]. These surfaces foul, but, because biological glues adhere poorly to them, are relatively easy to clean.

Fouling must be managed because it severely degrades the performance and energy costs of structures. Impacts range from cosmetic to increased costs for performance to impairment of performance to minor corrosion to compromised structural integrity due to corrosion [29] and mass of fouling build-up. Perhaps a good example of the impacts of fouling is nuclear power plants that cannot generate energy because their requirement for cooling water is compromised by organisms such as mussels which reduce flow into the plants, clog heat exchanges with juveniles, and cause corrosion due to their wastes. Engineers without biological backgrounds had no idea of biofouling and did not plan adequately for fouling in the generation of nuclear and conventional sea water cooled power plants around the world.

Because of the ability of biological molecules to interact with any surface, all surfaces foul. Because some biological propagules have only passive components, life settles on all surfaces. Thus, if one wants surfaces without appreciable life, locally toxic surfaces are necessary and even these surfaces foul. However, it is becoming increasingly clear that the use of broad-spectrum long-lived biocides, like those used in commercial fouling management hull coating systems, are not sustainable [2,7,30].

Since use of long-lived broad-spectrum biocides is not sustainable due to environmental degradation, what approaches might be sustainable? One option is the use of very short lived highly toxic biocides. Short half-life is an advantage because biocides do not build up in the environment, do not concentrate up the food chain, and have reduced effects on non-target species. Examples are the isothiazalones [30] and pyrithiones [31]. However, some organisms such as barnacles are relatively insensitive to these toxins, and single toxin solution coatings fail as toxin release rates fall [32], microbes adapt, and calcareous marofoulers provide non-toxic platforms on toxic surfaces for fouling community development.

## 3. Theoretical Construct

It is in the above, updated context that I thought about alternative strategies for fouling management. I developed a white paper and recruited colleagues that were interested. The initial team was Rittschof, Teo, and Maki. Our goal was environmentally benign fouling management. The team agreed to test my thoughts about evolution and how fouling organisms work in the context of off the shelf pharmaceuticals. If the ideas were correct, there were avenues for fouling management and intentional development of environmentally benign alternatives.

Evolution links all living organisms. DNA and RNA and the products of protein synthesis are evidence of the links. In our fascination for differences between organisms, we often overlook the generalities of evolution. At a slightly more detailed level, enzymes and control systems are conserved to the point that ancient enzymes like the serine proteases (trypsins and chymotrypsins) are found in all organisms. In fact, most organisms are more similar than they are different. They share biochemical parts, equipment, and pathways. For example, barnacles and humans, separated by about a billion years of evolution, share active sites of enzymes, sequences of proteins, hormones, neurotransmitters, and immune system components [9,33,34,35,36,37,38,39,40,41].

We started with the observation that juvenile and adult macro-fouling organisms are largely sessile, but that the dispersal/settlement stage is not. Although the settlement stage larvae of different macrofoulers are often visually very different, they are all roughly the same size [19] and all go through metamorphosis to the sessile life stage. Metamorphosis is a complex physiological process that requires extensive coordination. Metamorphosis is cued by sensory input followed by complex transduction events. For barnacles, this process is comparable to the event required to turn a pupa into a butterfly. Our hypothesis was that similar pathways coordinated metamorphosis in many macrofoulers. We started with barnacles and settlement using established bioassays [32,42,43,44]. Nowadays, there are many assays to choose from, for example [45].

We assumed transduction pathways were general physiological communication pathways, some of which could be altered by common pharmaceuticals. We also assumed the transduction pathways that direct metamorphosis in macro-fouling organisms are conserved. We hypothesized that (1) pharmaceuticals with known mechanisms of action in vertebrates, including man, could be used to disrupt metamorphosis transduction pathways in invertebrates; and (2) pharmaceuticals that were biologically active in vertebrates and invertebrates could also be bacteriostatic or bactericidal. We chose pharmaceuticals because they have known synthesis, known mechanisms of action in vertebrates, and known breakdown pathways [46].

Our target was to find pharmaceuticals that disrupted settlement of our laboratory barnacle model Amphibalunus *(=Balanus) amphitrite. A. amphritrite* is a common global fouler in temperate and tropical regions. We began our tests with barnacles and marine bacteria with a plan to discover pharmaceuticals with low acute toxicity to larvae and high potency, of at least micrograms per mL, in inhibiting barnacle settlement. We were not concerned about a specific transduction pathway or the details of the process, just that settlement stage larvae of barnacles either did not glue down or did not complete metamorphosis. Compounds passing the first hurdle would be tested for activity with other larvae and tested to see if they were bacteriostatic or bactericidal.

Practically, high volume pharmaceuticals were attractive because they were already produced in large quantities. We began our investigations with a rapid laboratory survey of toxicity and settlement inhibition of barnacle larvae by commonly prescribed and used pharmaceuticals in Singapore. Again, our goal was low acute toxicity with simultaneous high potency in barnacle settlement inhibition. Our measure was the therapeutic ratio [43,47], the ratio of toxicity to settlement inhibition. A ratio of one or less than one indicated that toxicity and settlement inhibition were by similar mechanisms. A ratio much larger than one suggested that toxicity and settlement inhibition were by different mechanisms. Our initial surveys rapidly identified several groups of pharmaceuticals that had low acute toxicity and were potent settlement inhibitors. Assays with marine bacteria confirmed several of the most attractive candidates were also bactericidal or bacteriostatic. Results with larvae of other macrofouling organisms supported our initial hypothesis that transduction pathways were conserved. We chose several candidates for further study and looked at their molecular structure.

## 4. The Path Forward

**Figure 1:**
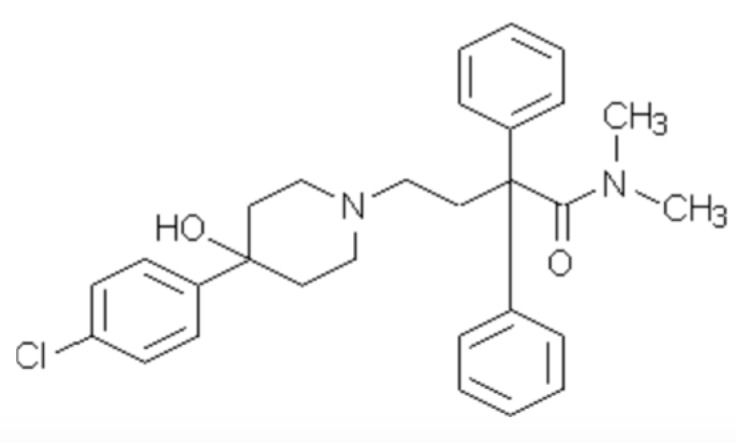
Imodium^®^.

We were elated that several pharmaceuticals met our criteria and supported all of our hypotheses [46,48]. One of the best candidates was Imodium^®^ **1** (Figure 1). One look at the structure and knowledge of how the drug works in the context of our goal of a short-lived molecule dictated our next research direction. Imodium^®^ is too large and too biologically stable. Therefore, we set out to simplify the molecule to reduce its half-life and reduce the generation of degradation products with unknown biological effects. This required expertise our group did not have. We visited a world class chemical engineering facility and were gratified when Christina Chia’s group including Felicity Jameson were interested.

**Figure 2:**
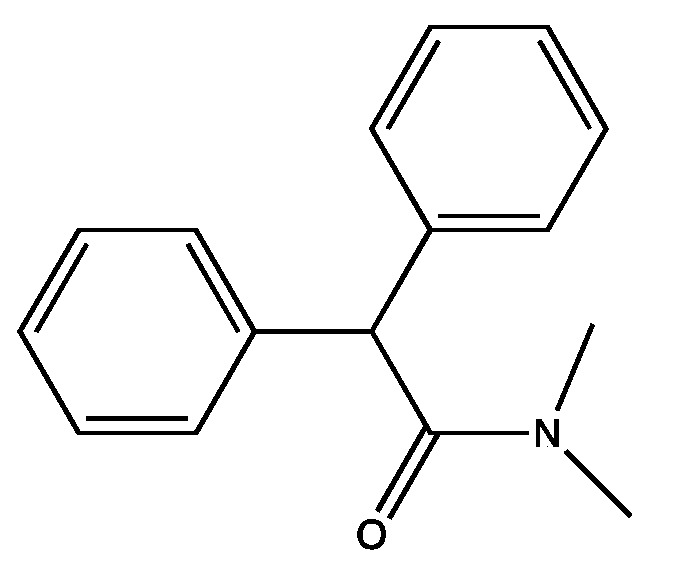
Active Pharmacophore.

First, we addressed structural changes that would modify the active molecule from one that is so refractory that it can be taken by mouth and impact the intestinal physiology of humans for several days to a molecule that is chemically simple and stable, but that in dilute form is degraded by bacteria. We began by looking for what we called the pharmacophore, a smaller molecule that was biologically active. We split the Imodium^®^ molecule in half and tested both halves. Surprisingly, both halves were biologically active. One half was much more attractive because it contained a nitrogen in an amide bond, several rings, and no halogens. That half (Figure 2) was as active as the original compound [49].

**Figure 3:**
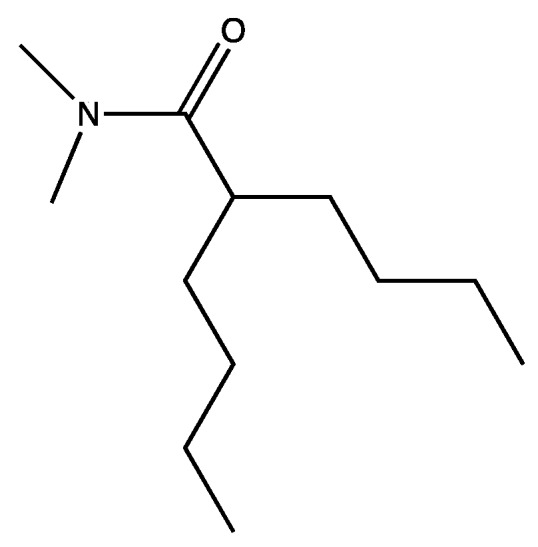
The two-legged man.

Molecule **2**, though much smaller than Imodium^®^, was still problematic because of its very stable rings. Rings are often steroidogenic, carcinogenic, and break down very slowly. Fortuitously, we had a clue as to how to modify the molecule. Imodium^®^ in vertebrates is an agonist for peptide receptors. Structure function studies with peptide pheromone receptors several decades earlier [50] provided the experimental and theoretical basis for structural modifications to maximize biological degradation while maintaining biological potency. Our talented synthetic chemists generated a quantitative structure function series that could be tested for toxicity, settlement inhibition, bacteriostatic activity, bactericidal activity, and biological breakdown. This huge and elegant series of experiments yielded a large number of options. We choose (Figure 3), which retained its biological activity and which we called the two-legged man.

**Figure 4:**
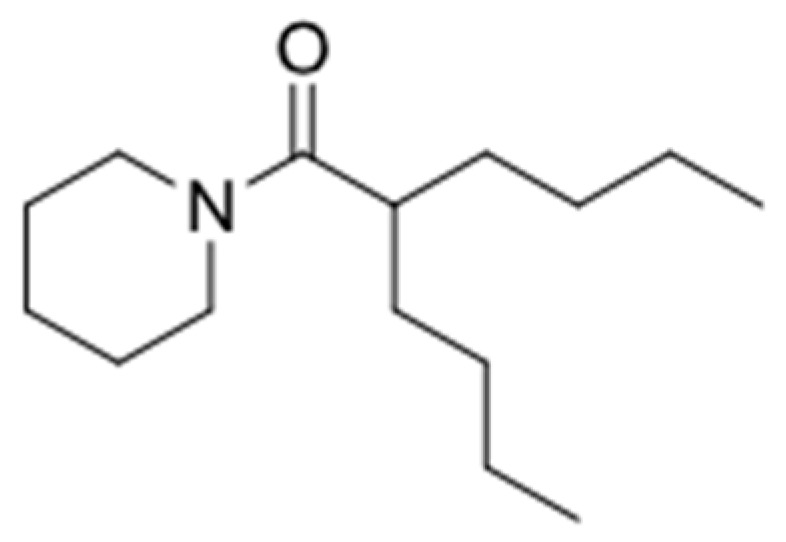
The practical pharmacophore.

In the two-legged man, we replaced each aromatic ring with a four carbon chain. In 1993, we published that four carbon chains were as potent as aromatic ring replacements in crab larval release peptide pheromone mimics [51]. The simplest molecule we could make that retained activity was a “one-legged man”. However, the one-legged man was an oil and was unattractive because we had no place on the molecule where we could modify and retain activity, other than by adding back the second leg. After a lot of additional experiments, the practical solution which satisfied everyone on our team was Figure 4.

**Figure 5:**
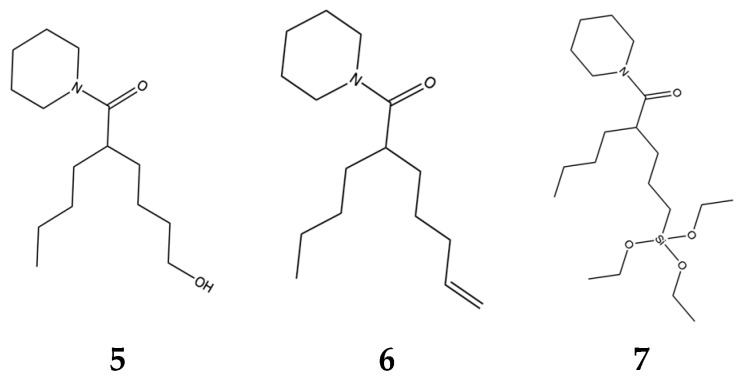
Molecules with “handles”.

The molecule in Figure 4 was practical because we could further modify it predictably. The goal of further modification was to provide a “handle” that could be used to enable us to couple the active molecule to delivery vehicles and then recover it through hydrolysis. We made a series of molecules **5**, **6**, **7** (Figure 5) with handles which were biologically active.

The molecules depicted above as well as many others are the subject of a patent that has been issued in many countries including China and the United States [52]. The handles are important because they enable active molecules to be incorporated into polymers. The silane modification is particularly attractive because the active ingredient can be coupled through an ester linkage to colloidal silica. Ester linkages to the hydroxyl groups on glass are pH sensitive. We have learned from the industry that developed silane derivatized silica gel for HPLC that there are measureable rates of hydrolysis of these linkages above pH 7. This is convenient when one considers the pH of sea water is approximately 8. Release of the active ingredient through hydrolysis of silane ester linkages is a viable option for delivery of active molecules from coatings. Using HPLC and associated detection technologies also provides sophisticated tools for tuning loading and release rates of molecules from glass particles that are already standard additives in satin and matte finish paints.

**Figure 6:**
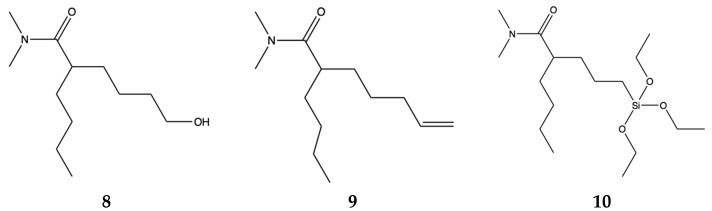
Biodegradable pharmacophores that can be coupled to other organic polymers **8**, **9** and coupled to silica **10**.

To bring this conversation full circle to our starting point, developing environmentally benign additives for fouling management, we still need to maximize the breakdown and make molecular changes of the pharmacophore for compatibility with different polymers used in hull coatings systems. The minimum components of the pharmacophore, reducing the six membered ring to two methyl groups on the nitrogen, are shown in three molecular structures that can be coupled for delivery **8**, **9**, **10** (Figure 6).

**Figure 7:**
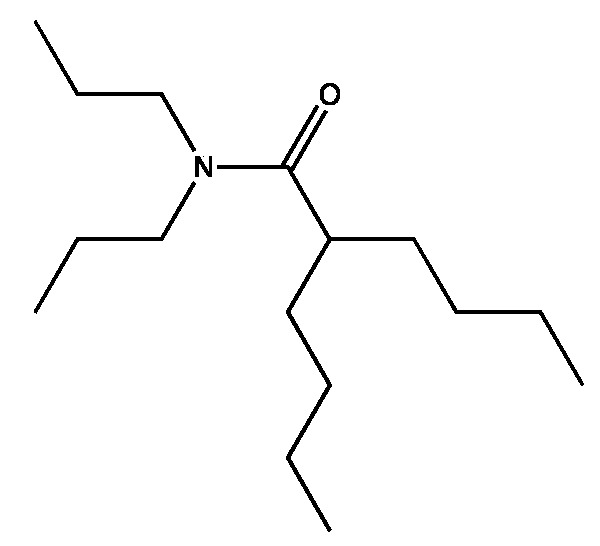
An untested, relatively easy to degrade version of the two-legged man.

From working with biological assays with larvae and bioactive molecules, there is an ideal balance of lipophilicity and hydrophilicity where especially amphipathic molecules are most potent. The functional relationship is convoluted but includes water solubility, surface activity, ability to enter organisms and cells, ability to interact with receptors, enzymes, or alter membranes, and ability to be consumed by bacteria. For the pharmacophore stripped of the engineering that makes it stable, this translates literally to modification of just a few carbons before the activity changes dramatically. A potent version is (4), the two-legged man. Opening the ring (Figure 7) maintains activity while rendering the pharmacophore slightly easier for microbes to degrade. This particular structure has not been tested for biological activity, but based upon the vast number of molecules we have tested it should be biologically active.

## 5. Business Context

Biofouling is pervasive. Management is necessary. Examples are teeth, contact lenses, implants, stents, sensors, oil pipelines, and any submerged surface including ship hulls. Management solutions can be for a niche market, for example to maintain the functions of submerged sensors, or for a large market share, like ship hulls or roofing materials. The smaller the niche (with the exception of biomedical devices and products used on and in humans), the less important are considerations of environmental impacts and sustainability. The molecules described above are attractive for fouling management because they are short-lived, not likely to accumulate or bio-concentrate, and they become simple biologically inactive molecules when the pharmacophore degrades.

These pharmacophores are particularly attractive when considered as part of a fouling management/anticorrosive system in which the anticorrosive coating (e.g., graphene), rather than being sacrificial, is a barrier to water and ions. However, graphene is a conductor which obviates the use of copper ions for fouling management because the cathodic protection used to protect exposed structures from corrosion prevents the release of copper ions. Disabling of fouling protection by suppressing the release of copper ions by cathode protection has been observed for water based yacht coatings in California.

Long studied by industry but a relatively new area for academia is compound antifouling/foul release coatings based on the ability to add oils and other compounds to the free space in polymers [44,53,54]. Though by industry standards these surfaces are inert, it is clear that compounds move to the surface, enter organisms [44,55], and interfere with the curing of biological glues and enzymes [56]. This is an exciting option for short lived pharmacophore delivery, but requires assessment of the consequences of leachates [57].

The final step prior to commercialization is a functional fouling management coating that meets national and international regulations. At this point, the final step is virtually impossible in the developed world. Registration of the biocide, which is what a pharmacophore must be considered, is slow and costly. Using Sea 9-211 as an example, registration in the United States cost about 11 million dollars and a decade. Registration in the EU was several hundred thousand dollars and several years [2]. It is clear that government policies shield existing technologies from new technologies and upfront cost maintains the status quo. However, regional development of new technologies in countries lacking the exiting fouling management infrastructure could be the first step in gaining a foothold in the business by providing the funds and data necessary to move novel approaches forward. However, this avenue has risks because regulations that protect and ensure thorough vetting are lacking. The future could be in a hybrid, where development is where business can be nimble and globalization requires thorough vetting of the safety of new technology. As globalization continues and wealth shifts around the globe, novel and sustainable technologies will become available and alter industry much as Uber and other web based transport options have impacted the taxi industry.

## 6. Conclusions

In this essay, I have provided the logic and story of a 15-year journey toward a sustainable fouling management strategy. The original premise was that highly conserved pathways that direct major life changes like metamorphosis in most organisms are points of attack for management strategies. The premise is substantiated and could provide an efficient way to search for future fouling management additives. It is also clear that a truly academic approach which fleshes out every detail is unnecessary. Although one does not need to know every biological detail, one can benefit from experience as demonstrated by the de-engineering steps which actually take advantage of fundamental principles that govern how molecules interact. This set of concepts has potential in pharmaceuticals as well as the design of additives for fouling management. Finally, if one has outstanding synthetic chemists, rapid production bioassays, and the ability to respect and communicate across disciplines, one can replace understanding and knowledge with empiricism. Eventually, even the strongest scholars run out of understanding and must resort to empiricism, which by default results in the development of new theories and new ways of understanding.

The value of this story is in the perspective it provides with respect to most of the issues necessary to develop an environmentally sustainable fouling management coating. This is not the only example, or the best. The interested person could look into the details of the Sea 9-211 story, the pyrithione story, and especially the metomadine story [33] because these are all commercial enterprises.
